# Spatial Patterns of Heat-Related Cardiovascular Mortality in the Czech Republic

**DOI:** 10.3390/ijerph13030284

**Published:** 2016-03-04

**Authors:** Aleš Urban, Katrin Burkart, Jan Kyselý, Christian Schuster, Eva Plavcová, Hana Hanzlíková, Petr Štěpánek, Tobia Lakes

**Affiliations:** 1 Institute of Atmospheric Physics, Czech Academy of Sciences, Boční II 1401, 14131 Prague 4, Czech Republic; kysely@ufa.cas.cz (J.K.); plavcova@ufa.cas.cz (E.P.); hanzlikova@ig.cas.cz (H.H.); 2 Faculty of Science, Charles University, Albertov 6, 12843 Prague 2, Czech Republic; 3 Department of Environmental Health Science, Mailman School of Public Health, Columbia University, 722 W 168th Street, New York, NY 10032, USA; burkarka@cms.hu-berlin.de; 4 Faculty of Environmental Sciences, Czech University of Life Sciences, Kamýcká 129, 16521 Prague 6, Czech Republic; 5 Global Change Research Centre, Czech Academy of Sciences, Bělidla 986, 60300 Brno, Czech Republic; petrstep2@gmail.com; 6 Department of Geography, Geoinformation Science Lab, Humboldt-Universität zu Berlin, Unter den Linden 6, 10099 Berlin, Germany; christian.schuster@hu-berlin.de (C.S.); tobia.lakes@geo.hu-berlin.de (T.L.); 7 Institute of Geophysics, Czech Academy of Sciences, Boční II 1401, 14131 Prague 4, Czech Republic; 8 Czech Hydrometeorological Institute, Regional Office Brno, Kroftova 2578, 61667 Brno, Czech Republic

**Keywords:** heat stress, mortality, socioeconomic status, spatial differences, cardiovascular disease

## Abstract

The study examines spatial patterns of effects of high temperature extremes on cardiovascular mortality in the Czech Republic at a district level during 1994–2009. Daily baseline mortality for each district was determined using a single location-stratified generalized additive model. Mean relative deviations of mortality from the baseline were calculated on days exceeding the 90th percentile of mean daily temperature in summer, and they were correlated with selected demographic, socioeconomic, and physical-environmental variables for the districts. Groups of districts with similar characteristics were identified according to socioeconomic status and urbanization level in order to provide a more general picture than possible on the district level. We evaluated lagged patterns of excess mortality after hot spell occurrences in: (i) urban areas *vs.* predominantly rural areas; and (ii) regions with different overall socioeconomic level. Our findings suggest that climatic conditions, altitude, and urbanization generally affect the spatial distribution of districts with the highest excess cardiovascular mortality, while socioeconomic status did not show a significant effect in the analysis across the Czech Republic as a whole. Only within deprived populations, socioeconomic status played a relevant role as well. After taking into account lagged effects of temperature on excess mortality, we found that the effect of hot spells was significant in highly urbanized regions, while most excess deaths in rural districts may be attributed to harvesting effects.

## 1. Introduction

Harmful effects of high atmospheric temperature on human health and lives have been documented in mid-latitudes [1,2] as well as in tropical climates [3,4]. Due to the rise in temperature with climate change, increased frequency and magnitude of extreme heat events are projected for the 21st century in comparison to recent climatic conditions [5,6,7]. Therefore, increased heat-related mortality is expected in future [2,8]. On the other hand, studies have documented declining impact of heat waves in developed countries due to behavioural and technological adaptation [9,10,11], including more effective public warning and response [12]. Therefore, uncertainty in the projection of temperature-related mortality is still large [2,8,13,14].

Young children, the elderly and persons in poor physical and medical condition [15,16], and especially those with cardiovascular and respiratory diseases, are most affected by heat stress [1,17,18]. In the Czech Republic, excess all-cause and cardiovascular mortality due to hot conditions have been observed in several studies that considered the population as a whole [19,20]. The elderly, women and people with chronic cardiovascular diseases (CVDs) were found to be the populations groups with the largest heat-related mortality [21,22,23].

Many studies have investigated the effects of heat waves on urban populations, which are those most affected by heat-related mortality [24,25,26]. a higher percentage of elderly, socially deprived and isolated populations (people living alone, immigrants, ethnic minorities) have been identified as factors increasing heat-related mortality [27,28,29]. In addition to socioeconomic factors, such land cover characteristics as the amount of impervious surface, green space, and unvegetated areas in the neighbourhood have been associated with increased heat vulnerability [27,30,31,32] and heat-related mortality [29,33,34]. Heat stress has nevertheless been found to be a significant risk also for the rural population [27,35,36,37]. Analyses in the Czech Republic have revealed comparably short-term response of cardiovascular mortality in both urban and rural population regardless of what temperature measure was used [38,39]. Spatial patterns in temperature-health relationships taking socioeconomic and environmental factors into account have not yet been investigated in the Czech Republic, however.

During heat waves, excess mortality often occurs for several days which are then followed by a decrease of mortality rates to below expected levels (so-called “mortality displacement” or “harvesting”; [40,41,42]). Although magnitudes of the short-term mortality displacement have been observed to vary considerably among populations of different cities [1,43,44,45,46], studies assessing spatial variability in heat-related mortality according to urban *vs.* rural and socioeconomic differences in a specific geographic area do not usually address the harvesting issue (including [38,39]).

In this study, we investigate spatial as well as temporal patterns for the effects of high temperature extremes on cardiovascular mortality in the Czech Republic at a district level. Effects of demographic, socioeconomic, and physical–environmental factors on excess cardiovascular mortality in 76 administrative districts are identified. Groups of districts with similar characteristics are compared in order to provide a more general picture and to evaluate different temporal patterns of excess mortality after hot spell occurrence in (i) urban areas *vs.* predominantly rural areas, and (ii) regions with different overall socioeconomic level.

## 2. Data and Methods

### 2.1. Study Area

**Figure 1:**
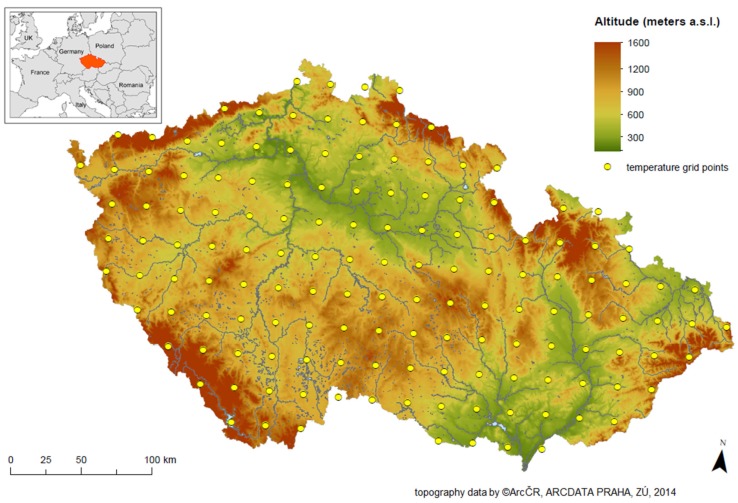
Topography of the Czech Republic (Digital Elevation Model provided by ARCDATA PRAHA) and distribution of regular temperature grid points (GriSt data set as described in [49]).

The Czech Republic is a Central European country with a temperate climate and relatively varied landscape (Figure 1). Its approximately 10.2 million total inhabitants (as of 2001, with only minor changes during 1994–2009) are distributed into 77 districts (*Nomenclature of Units for Territorial Statistics* level 4 (NUTS 4) according to EUROSTAT [47]), ranging from 42,000 inhabitants in the Jeseník District to about 1.2 million inhabitants in the capital city of Prague. While our mortality data start in 1994, the Jeseník District (CZ0711—Czech Statistical Office coding [48]) was established in 1996 by its separation from the Šumperk District. In order to retain homogeneity in mortality data, we considered Jeseník and Šumperk as a single district in our study (CZ0715). Thus, we analysed 76 districts.

### 2.2. Meteorological Data

**Figure 2:**
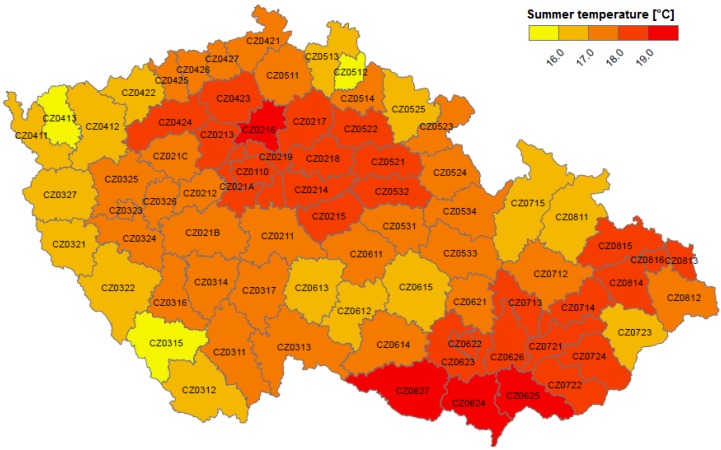
Average summer temperature (June–August 1994–2009) in districts of the Czech Republic calculated from the GriSt data set. Districts are labelled according to the Czech Statistical Office coding [48].

We considered average daily temperature in each district as a proxy variable for ambient thermal conditions. For its calculation, we used a high-resolution regular temperature grid covering the whole of the Czech Republic (see Figure 1). The GriSt data set with resolution 25 × 25 km is based on interpolated mean daily temperature data from irregularly spaced meteorological stations operated by the Czech Hydrometeorological Institute as described in Kyselý and Plavcová [49]. Such data were not available for other meteorological variables needed as inputs for calculating biometeorological indices (see Błażejczyk *et al.* [50]). Every grid box provides information about mean daily temperature from 1 January 1994 to 31 December 2009 and average altitude. Total population in each grid box in 2001 was calculated from the Gridded Population of the World (v3) [51]. Finally, the average daily temperature in each district was calculated from grids falling into the district area weighted by population within these grids (Figure 2). Average altitude of grids falling into each district was calculated as well. Zonal statistics tools in QGIS Desktop 2.4.0 were used for transformation of the grid data to districts.

### 2.3. Mortality Data

We analysed daily mortality from cardiovascular diseases (CVDs; defined as codes I00–I99 according to the International Statistical Classification of Diseases, 10th Revision [ICD-10]) in each district during 1994–2009. Mortality from CVDs comprises more than 50% of total mortality in the Czech Republic with a total record of 930,659 deaths occurring during the study period [52]. As the population count and structure vary distinctly between districts, daily mortality rates were calculated by the direct standardization procedure, using the mid-year population of each district in the Czech Republic and the standard WHO European population as the standard [53]. Daily baseline mortality for individual districts was determined using a single location-stratified generalized additive model (“mgcv” package in R (version 2.15.2)) [54]. A spline function with seven degrees of freedom (df) per year (total df = 112) was used according to Bhaskaran *et al.* [55], in order to take account for long-term trend and seasonality. Additional components taking account for the day of week and the district were defined by categorical variables. Mean relative deviations of mortality from the baseline (excess mortality) on days with average temperature above the 90th percentile (hot days) in summer seasons (June–August) 1994–2009 were calculated for all districts (*DevCVD*). The 90th percentile instead of the 95th percentile was chosen because it yields larger sample size, which is beneficial for the analysis at district level. Statistical significance of mean relative deviations was evaluated by comparison with the 90% confidence bounds around the zero line, estimated from the 5% and 95% quantiles of a distribution calculated by the Monte Carlo method (*cf.* Plavcová and Kyselý [56]). Spatial autocorrelation of mortality deviations on all summer days as well as on hot days was tested by Global Moran’s *I* criteria [57] in ArcGIS 10 software.

### 2.4. Analysis of Spatial Patterns of Heat-Related Mortality

We used demographic and socioeconomic characteristics from the Census 2001 database (provided by the Czech Statistical Office), land cover characteristics from the CORINE land cover 2000 database (the Czech Environmental Information Agency), and mean summer temperature (*summer T*) and mean *altitude* calculated from the GriSt data set in order to identify effects of demographic, socioeconomic, and physical–environmental factors on spatial differences in heat-related mortality. The selection of demographic and socioeconomic variables was based on a literature review [30,58,59,60,61] and adapted with respect to the limitations of the Census database. We applied a modified population density variable according to the OECD’s international terminology [62] (hereafter termed *OECD* criterion) which defines a level of urbanization by percentage of inhabitants living in municipalities with population density less than 150 inhabitants per 1 km^2^. An index of socioeconomic status (*SES*) for each district was calculated as a sum of *z*-scores for 3 factors of social deprivation related to elevated mortality in the Czech Republic [63]. Percentages of unemployed population (*% unemployed*), people without secondary school diploma (*% low education*), and single-person households (*% singles*) were included into the index. The final index is a dimensionless variable with a range between 2.66 (highest *SES*) and −5.88 (lowest *SES*) across the districts in the Czech Republic.

Land cover structure of each district was calculated using zonal statistics tools in QGIS Desktop 2.4.0. Percentage of impervious surface (*% impervious*) representing residential, industrial, commercial and transport areas was chosen similarly to other studies as a land cover variable enhancing heat stress [27,28,29,36,37,61,64]. The specific list of all variables used is presented in Table 1. Spearman’s correlation coefficients (and their *p*-values) of relationships between mean relative mortality deviations on hot days and independent variables were calculated using the “rcorr” function in the R package “Hmisc” (version 3.14-3) [65]. To check for multicollinearity between explanatory variables, we used variance inflation factors (VIFs) calculated in the ordinary least squares (OLS) regression in ArcGIS. If two or more explanatory variables had a large VIF (>7.5), the variable leading to the OLS regression model with lower percentage of variance explained was excluded from further analysis. After reduction of variables, we performed a bidirectional stepwise regression procedure testing all remaining explanatory variables, in order to identify the most significant variables affecting excess CVD mortality due to heat. The “stepAIC” function in the “MASS” package in R (version 2.15.2) adds or drops variables to or from the model repeatedly, fits those models, and computes a table of the changes in fit according to the Akaike information criterion (AIC) and amount of variance explained [66], *i.e.*, the model with the lowest AIC and all significant parameters (as determined by an F test, *p* < 0.05) was selected. Spatial autocorrelation of the model residuals was evaluated with Global Moran’s *I* criteria. Ultimately, linear relationships of CVD mortality deviations with independent variables representing socioeconomic status (*SES*), population density (*OECD* criterion), and environmental effects (*summer T*) were examined individually for districts with *low* (<−0.50 StdDev), *intermediate*, and *high* (>0.50 StdDev) *SES*.

### 2.5. Analysis of Temporal Patterns of Heat-Related Mortality in Groups of Districts

**Figure 3:**
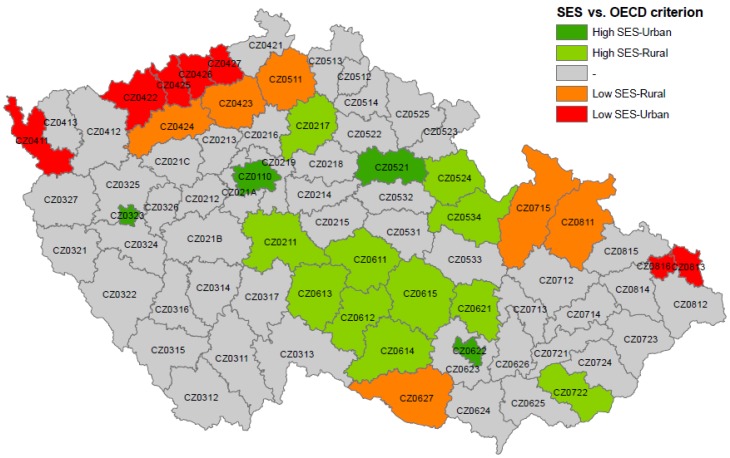
Four *groups of districts* identified according to *high* (>0.50 StdDev) and *low* (<−0.50 StdDev) index of socioeconomic status (*SES*) and *urban* (*OECD* criterion <25%) and *rural* (*OECD* criterion > 37.5%) population.

*Groups of districts* with similar characteristics were defined in order to obtain greater population sample sizes and provide a more general picture of results on the district level. The districts with *high SES* and *low SES* were split into *urban* (*OECD* < 25%) and *rural* (*OECD* > 37.5%) districts according to the *OECD* criterion of the population density calculated for the Czech Republic by Blatecká [67]. Mortality rates and temperature data of all districts in each of the four groups (*high SES-Urban*, *high SES-Rural*, *low SES-Urban*, and *low SES-Rural*) were averaged and baseline mortality for each group using the same GAM formula as for individual districts was again calculated. The four *groups of districts* are displayed in Figure 3 and their characteristics are presented in Table 2.

Hot spells were defined as at least two consecutive days with average temperature above the 90th percentile of its distribution in summer seasons (June–August) 1994–2009 in a given *group of districts*. A percentile definition was chosen because it leads to similar numbers of days in hot spells in each group of districts. Relative deviations of CVD mortality from the baseline on days D − 2 (2 days before the beginning of a hot spell) up to D + 14 were averaged over all hot spells in order to assess lagged patterns of mortality deviations. Statistical significance of mean relative deviations was evaluated by comparison with the 90% and 95% confidence bounds around the zero line, estimated from the 2.5%, 5%, 95% and 97.5% quantiles of a distribution calculated by the Monte Carlo method (*cf.* Plavcová and Kyselý [56]). As the effect of a heat event on significantly elevated mortality usually occurs up to 3 days after the event [1,19,42], cumulative excess CVD mortality on days D + 0 to D + 3 and D + 4 to D + 14 after a hot spell’s onset was compared in order to estimate magnitude of the mortality displacement effect.

## 3. Results

### 3.1. Heat-Related Mortality in the Czech Republic at District Level

**Figure 4:**
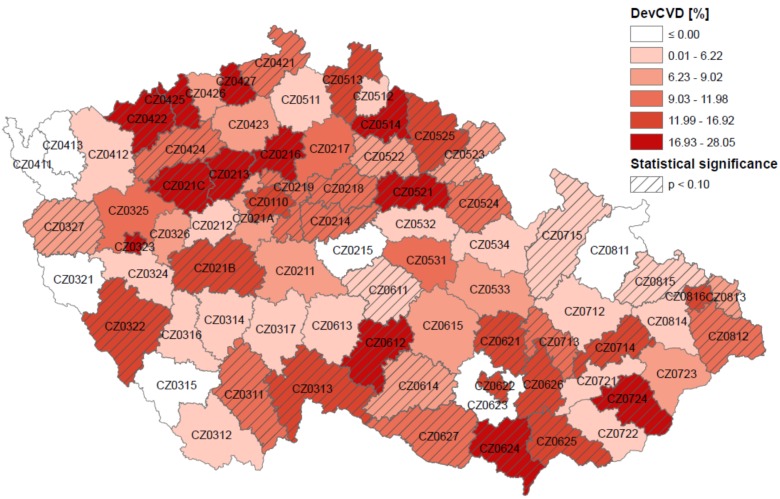
Spatial distribution of mean relative mortality deviations due to CVDs on hot days (*DevCVD*). Districts are labelled according to the Czech Statistical Office coding [48].

About 148 hot days were defined in each district during 1994–2009. The highest and statistically significant (*p* < 0.1) heat-related mortality (*DevCVD*) was generally experienced in the northwest and southeast Czech Republic and in large municipalities (Prague-code CZ0110, Pilsen-CZ0323, Brno-CZ0622, Ostrava-CZ0816; Figure 4). These areas are mostly located in the lowest and warmest parts of the Czech Republic (*cf.* Figure 1 and Figure 2). However, the test for spatial autocorrelation (Global Moran’s *I*) did not show any significant spatial clustering of mean mortality deviations on all summer days as well as on hot days.

### 3.2. Explaining Spatial Patterns of Heat-Related Mortality

Spearman’s correlation analysis revealed generally very weak and insignificant correlation of relative CVD mortality deviations (*DevCVD*) with most of the socioeconomic factors (Table 3). On the contrary, variables representing effects of physical environment and urbanization level (*altitude*, *summer T*, and *% impervious*) correlated significantly (*p* < 0.05) with mortality anomalies on hot days (*r* = 0.37, −0.37, and 0.34 respectively), although correlation coefficients for the significant variables were still rather weak. Variables correlating significantly with mortality deviations correlated also significantly with one another. In particular, *altitude* and *summer T* showed strong negative correlation (*r* = −0.92). These two variables were also associated with large VIFs (>7.5) which indicates their strong collinearity. Since *summer T* led to higher percentage of variance explained, *altitude* was excluded from the regression analysis.

The stepwise regression procedure identified district’s *summer T* as the most significant factor positively linked to excess mortality (Table 4). The increased population density (decreased *OECD* criterion) was chosen by the model as the second variable significantly associated with increased mortality. Global Moran’s *I* criteria close to 0 indicated no spatial autocorrelation of residuals from the stepwise regression model.

**Figure 5:**
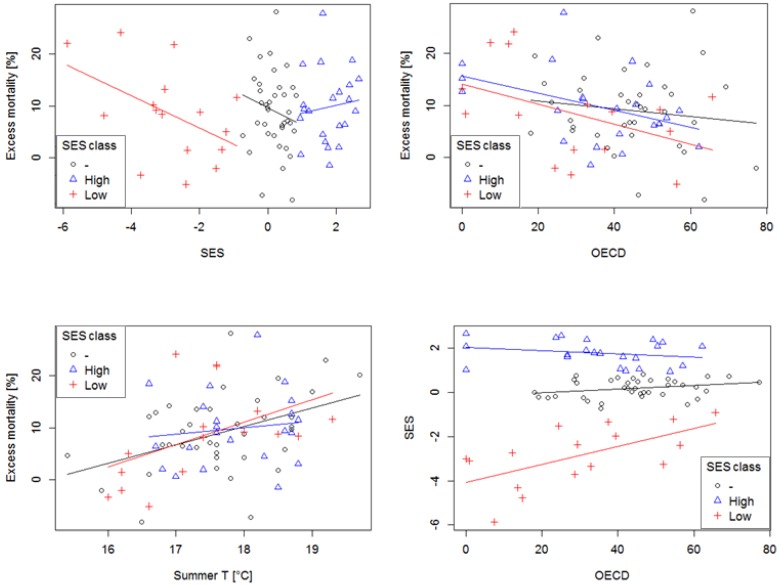
Scatterplots comparing mean relative excess mortality due to CVDs on hot days (*DevCVD*) with index of socioeconomic status (*SES*), percentage of rural population (*OECD* criterion), and mean summer temperature (*Summer t*) for districts with *low* (<−0.50 StdDev), *intermediate* (-), and *high* (>0.50 StdDev) socioeconomic status (*SES*). Coefficients of regression lines are reported in Table 5. See Table 1 for description of variables examined.

In the analyses for all districts considered together, regression procedure did not reveal any significant effect of socioeconomic status (*SES*) on heat-related mortality (*DevCVD*). However, regression analysis between *DevCVD* and selected explanatory variables showed significant changes in the relationships (Table 5, Figure 5) when performed individually for districts with *low*, *intermediate* and *high SES*. In *high SES* districts, the decreased *OECD* criterion (*i.e.*, increased percentage of urban population) was the only variable significantly (*p* < 0.1) linked to increase in *DevCVD*. In the largest group of districts with the *intermediate SES*, only increased *summer T* was significantly (*p* < 0.05) associated with increased *DevCVD*. But in districts with *low SES*, increased *DevCVD* was significantly (*p* < 0.05) linked to increased *summer T*, decreased *OECD* criterion as well as decreased *SES* (considered as explanatory variable). Decreased *SES* was also significantly related to a decrease of the *OECD* criterion in the same category.

Results obtained from the analysis at the district level gave us general information about spatial distribution of heat-related excess mortality due to CVDs. Although in most districts the mean mortality deviations were positive and significantly different from zero (Figure 4), numbers of cases especially in rural districts were rather low and baseline estimates in such districts were affected by overdispersion. Therefore, the districts were divided into *groups of districts* according to the *SES* index and the *OECD* criterion in order to compare effects of high temperature in larger population samples.

### 3.3. Explaining Lagged Patterns of Heat-Related Mortality in Groups of Districts

**Figure 6:**
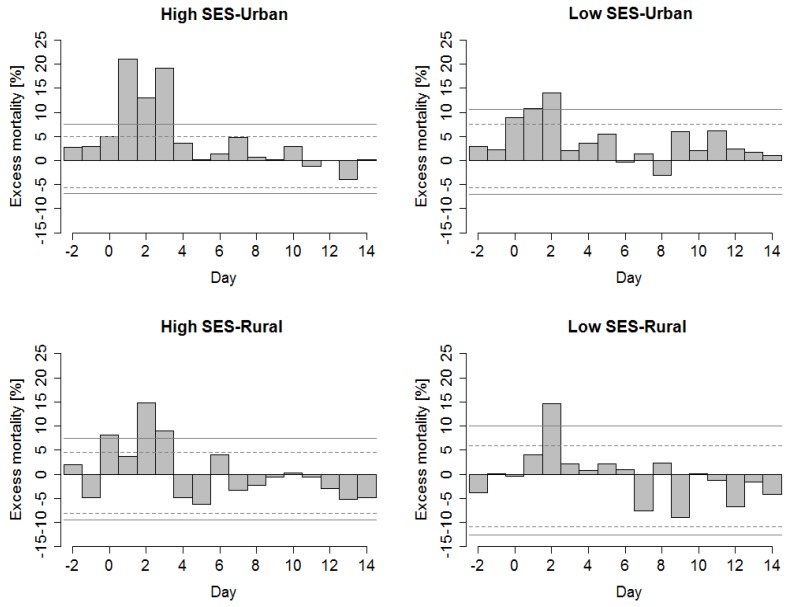
Mean relative excess mortality due to CVDs on days D − 2 to D + 14 around a hot spell’s onset in the *groups of districts*. Confidence bounds around the zero line are indicated by dashed (90%) and solid (95%) lines, respectively.

**Figure 7:**
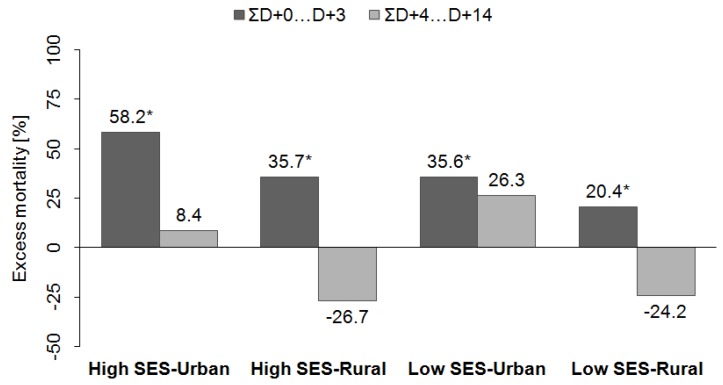
Cumulative excess mortality (relative to the daily baseline mortality) due to CVDs on days D + 0 to D + 3 (ƩD + 0…D + 3) and D + 4 to D + 14 (ƩD + 4…D + 14) after a hot spell’s onset in the *groups of districts*. * denotes mean cumulative excess mortality above the 95% quantile of a distribution calculated by the Monte Carlo method.

About 35 hot spells were defined in each *group of districts* during 1994–2009. In all groups, significant excess mortality occurred between days D + 0 and D + 3 after a hot spell’s onset followed by a decline in mortality deviations (Figure 6). While in rural districts the lagged mortality deviations (D + 4 and later) were predominantly negative, in urban districts they were rather positive during the whole two-week period after a hot spell’s onset. When cumulative mortality deviations on days D + 0 to D + 3 and D + 4 to D + 14 after a hot spell’s onset were compared (Figure 7), the immediate cumulative effect (ƩD + 0…D + 3) was largest in the most densely populated and the warmest *group of districts* with high socioeconomic status (*high SES-Urban*, 58.2% of daily mortality), while it was lowest in the least populated group with low socioeconomic status (*low SES-Rural*, 20.4%). When taking into account the lagged effects (ƩD + 4…D + 14), however, we observed negative relative mortality deviations in rural *groups of districts* which results in total heat-effects during the two-week period close to zero (8.9% and −3.8% in *high SES-Rural* and *low SES-Rural*, respectively). On the contrary, the cumulative lagged effects were positive in urban districts. In the urban district with low *SES* (*low SES-Urban*), the lagged effect was comparable with the immediate one (26.3%), while in the *high SES-Urban* group the additional increase in excess mortality was rather small (8.4%).

## 4. Discussion

We analysed spatial and temporal patterns of links between daily CVD mortality and mean daily temperature during summer (June–August), 1994–2009 in the Czech Republic. We further evaluated these patterns with respect to demographic, socioeconomic, and physical-environmental differences. The largest relative mortality deviations on days that exceeded the 90th percentile of summer temperature distribution (hot days) were generally observed in the warmest and most densely populated regions of the Czech Republic.

### 4.1. Spatial Patterns of Heat-Related Mortality

When all districts in the Czech Republic were considered together, characteristics of physical environment (mean summer temperature, altitude) and urbanization level (population density, percentage of impervious surface) had a significant influence on heat-related CVD mortality, while socioeconomic status did not show any significant effect. On the other hand, a comparison of districts with low and high index of socioeconomic status (*SES*) revealed that below a certain threshold, *SES* has a relevant influence on excess mortality.

Although previous studies have demonstrated differences in the heat–health relationship between different cities and different population groups, there are only few studies including also rural areas and considering socioeconomic and land cover factors affecting spatial differences in heat-related mortality. In the U.S., Sheridan and Dolney [35] found a comparable effect of heat on mortality in urban and rural counties in Ohio. In Massachusetts [60] and Georgia [36], demographic (percentage of elderly, percentage of African-Americans) and socioeconomic (social isolation, poverty) factors were more important than was the level of urbanization (as represented by population density and proportion of the impervious surface). Recently, Kovach *et al.* [37] in North Carolina found greater rates of heat-related illnesses in rural than urban locations, and a greater proportion of workers in agriculture with less access to air conditioning has been mentioned among the reasons of high heat-vulnerability in rural populations [35,37]. On the other hand, decreased vegetation, living in poverty, and low education were associated with increases in heat-related illnesses in urban locations [37]. However, excess morbidity and mortality due to heat in U.S. cities is reduced by the protective effect of air conditioning [35,68]. Air conditioning is much less widespread in Europe than in American households [69], and population in the most densely populated areas is usually most affected by heat in Europe [24,26]. Moreover, in contrast to the U.S. and many western-European countries, social differences between urban, suburban and rural populations in the Czech Republic are much smaller, as the country ranks as having among the lowest income inequality of OECD countries [70], and the lowest estimated percentage of population severely deprived with respect to health, education and/or standard of living in the European Union (the Human Poverty Index, HPI; [71]). In addition, none of the aforementioned studies considered the effects of physical–geographic conditions such as local climate and topography. Strong collinearity between districts’ average temperature and altitude found in our results suggests that highest heat-related mortality is generally observed in districts with low altitude, relatively hotter local climate and higher level of urbanization. Nevertheless, the significant relationship between decreased socioeconomic status and increased heat-related mortality in the most deprived district group highlights the role of social deprivation in vulnerability to heat across the population of the Czech Republic.

### 4.2. Lagged Patterns of Heat-Related Mortality

Another limitation of many studies assessing spatial variability in heat-related mortality within specific geographic areas is that they do not take into account the mortality displacement (harvesting effect) issue. Analysis of the four *groups of districts* with larger sample sizes allowed us to study lagged patterns of mortality deviations with more statistical power than for individual districts. While in urban districts the lagged sums of mortality deviations were still positive, we observed large decreases in cumulative relative mortality deviations in rural districts. A decline in mortality a few weeks after a heat wave due to mortality displacement has been documented in many studies [42,43,44,45,46]. Although estimates of the mortality displacement magnitude vary based on the methodology used and between different populations [1,44], our findings suggest that, at least in the Czech Republic, the effect of mortality displacement after hot spells is small and insignificant in urban districts, while most of the excess deaths in rural districts may be attributed to harvesting.

The highest short-term effect of hot spells in the *high SES-Urban* group suggests that high socioeconomic status was a risk factor of increased excess CVD mortality in the stratified analysis. However, the regression analysis at the district level showed *SES* as a relevant factor only in the *low SES* districts. Therefore, highest population count and density, highest average summer temperature due to low altitude, and generally worst thermal conditions in comparison to other *groups of districts* due to high proportion of artificial surface are probably the main reasons for the largest short-term effect of hot spells on excess CVD mortality in the *high SES-Urban* group. Many studies have documented strongest heat effects on mortality in the most densely populated areas [24,25,26] due to urban heat island effects [72,73], although this may be altered by large-scale factors such as altitude and topography [74] as well as such fine-scale factors as proportions of impervious, green or water surfaces [34,75,76,77].

Short-term heat effect was reduced in the urban *group of districts* with low socioeconomic status (*low SES-Urban*) in comparison to *high SES-Urban*. However, when the cumulative relative mortality deviations during the two-week period after a hot spell’s onset were considered, excess CVD mortality in *low SES-Urban* was comparable with that in the *high SES-Urban* group (62% and 67%, respectively). This may be caused not only by relatively high levels of urbanization but also by social deprivation of populations in *low SES-Urban* districts, inasmuch as regression analysis at the district level had shown a significant relationship between socioeconomic status and heat-related CVD mortality for districts with low socioeconomic status. Dzúrová [78] had demonstrated significant relationships between environmental deprivation, social deprivation, and poor health condition within the population of the Czech Republic. Long-term exposure to high concentrations of particulate matter (PM) has been associated with development of chronic diseases of the veins and heart [79,80,81]. People with chronic CVDs have been most affected by excess mortality during hot spells in the Czech Republic [22,38]. Highest concentrations of PM_10_ and O_3_ have been associated with oppressive weather in Prague, although they were not identified as significant factors linked to excess CVD mortality by a stepwise regression model within the oppressive air masses [82]. Since heavy industry and coal mining are typical economic activities in districts of the *low SES-Urban* group, poor health condition due to long-term exposure to high air pollution concentrations [83] and in general highest environmental deprivation within the Czech Republic in these districts may have a significant effect on lagged excess mortality due to CVDs during hot spells. However, inasmuch as air pollution characteristics are not spatially representative because they vary considerably across space, and causal relationships among temperature, air pollution, and human health are associated with large uncertainty [84], we did not consider the effect of air pollution in this study.

### 4.3. Limitations of the Study

A usual issue of studies assessing spatial variability of weather-mortality relationships is to find a trade-off between geographic specificity and sample size when selecting a unit of analysis [60]. The length of time period (number of days) and number of events (e.g., deaths) per day are factors determining statistical power and precision of time series modelling [55]. For example, Hondula and Barnett [85] employed a hierarchical Bayesian model treating each unit as not completely independent from every other. We applied a single generalized additive model stratifying the whole dataset to individual districts by a categorical variable, which allowed us to model baseline mortality consistently in all districts and helped to deal with small population counts. Mortality deviations in individual districts are still subject to great uncertainty, as numbers of deaths in districts of the Czech Republic show high variability and only a small percentage of total variance can be explained by meteorological factors [82]. Therefore larger regions should be considered for heat-related mortality assessment and biometeorological forecast (e.g., [86]) in the Czech Republic rather than individual districts.

Our findings also highlight the importance of relating heat risk factors to appropriate health variables when evaluating vulnerable populations and regions. This is an issue in particular for studies mapping heat vulnerability without validation on health outcomes [30,58,64]. As demonstrated by Reid *et al.* [31], a heat vulnerability index calculated from socioeconomic and environmental risk factors can be consistently associated with poor health on both normal and hot days. In our study, low socioeconomic status (*SES*) was strongly correlated with high mortality (not shown), while the association with excess mortality on hot days was weak. Excess deaths during a heat wave can be considered as an extreme outcome of heat stress. Previous studies have revealed no significant effect of high temperatures on hospitalizations due to CVDs [23,38,82,87]. Nevertheless, health outcomes such as emergency calls [27,88] and hospital admissions for respiratory and heat-related diseases [31,89] might be significantly related to high temperatures and more affected by socioeconomic differences than cardiovascular mortality.

Another limitation of this study is that the associations observed for data aggregated at district level may not necessarily represent associations existing at an individual level—the so-called ecological fallacy issue [90]. Similarly, the associations between mortality and explanatory variables observed for the whole group of districts may vary spatially, as demonstrated by the stratified analysis for districts with *low*, *intermediate* and *high SES*. However, as we did not find any significant spatial autocorrelation in the residuals from baseline mortality nor in residuals from the step-wise regression procedure, we did not use any spatially varying coefficient model.

Finally, the study may potentially be subject of the modifiable areal unit problem as all variables were aggregated to district spatial units. Repeating the study at different analysis level may produce variations in the results [91]. However, the results for the individual districts and for aggregated regions supported each other.

Another issue of this study is the use of gridded temperature data. As the spatial distribution of the grid is 25 × 25 km, it does not fully represent fine-scale thermal and environmental conditions in individual municipalities. This is particularly true in the *low SES-Urban* districts which are located in areas with large differences in topography and thermal conditions ranging from relatively large cities situated in a warm climatic region to surrounding mountain areas with cooler climate [92]. In order to take this issue into account, temperature data was weighted by population counts in every grid box when mean daily temperature in districts was calculated and percentiles were used instead of absolute temperature thresholds for the definition of hot days. Nevertheless, the effect of average summer temperature and average altitude on mortality deviations on hot days may be partly underestimated in the correlation and regression analyses. On the other hand, the gridded temperature data, representing spatial means rather than point values [49], allow capturing temperature conditions in individual districts better than raw station data or their averages.

## 5. Conclusions

In this study we analysed spatial patterns of links between high temperature extremes and daily cardiovascular mortality at a district level. We considered differences between districts’ demographics, socioeconomic status, as well as physical-environmental conditions.

The largest relative mortality deviations on hot days were generally observed in the warmest regions of the Czech Republic that involved most urbanized areas. We revealed significant effects of physical environment (altitude, mean summer temperature, artificial surface) and urbanization (population density) on excess cardiovascular mortality on hot days when all districts in the Czech Republic were considered together. The level of socioeconomic status played a relevant role only in the most deprived populations.

Analysis of mortality deviation patterns for larger groups of districts during two weeks after a hot spell’s onset revealed no mortality displacement in large municipalities, while most of the excess deaths in rural districts may be attributed to harvesting. Highest population count and density, highest average temperature due to low altitude, and generally worst thermal conditions due to high proportion of artificial surface were the factors associated with largest excess cardiovascular mortality due to hot spells. In addition, long-term exposure to highest environmental and socioeconomic deprivation within the Czech Republic in urban districts with low socioeconomic status may have a significant effect on lagged excess mortality associated with hot spells due to the higher percentage of people with chronic cardiovascular disease. Inasmuch as coal mining and associated heavy industry (energetics, metallurgy) are typical economic activities in these districts, their populations are potentially at risk of increasing social deprivation in future due to a decline of mining and industrial transformation. The significant relationship between decreased socioeconomic status and increased heat-related mortality within the most deprived district group highlights the role of socioeconomic status in adaptability to heat across the population.

We identified districts and regions in the Czech Republic whose populations are most at risk of excess mortality due to heat stress and might be at increasing risk in the future. Our results are potentially useful for better targeting biometeorological forecasts and warnings, although larger regions rather than individual districts need to be considered to achieve a reasonable statistical power. Such other health outcomes as emergency calls and hospital admissions for respiratory and heat-related diseases should be considered in follow-up research regarding the effects of heat stress on public health in the Czech Republic.

## Figures and Tables

**Table 1:** District characteristics tested in the analysis.

Variable	Description
DevCVD	Mean relative CVD mortality deviations on hot days with average temperature above the 90th percentile in June–August 1994–2009
SES	Index of socioeconomic status; sum of z-scores for % low education, % unemployed and % singles
% elderly	% of population older than 65 years
% low education	% of population without secondary school diploma
% unemployed	% of unemployed population
% singles	% of single-person households
OECD	% of inhabitants in municipalities with population density less than 150 inhabitants/km^2^
Summer T (°C)	Mean summer (1 June–31 August 1994–2009) temperature in °C
Altitude (m a.s.l.)	Average altitude in metres above sea level
% impervious	% of impervious surface area (categories 1.1 and 1.2 in the CORINE land cover classification)

**Table 2:** Total population and average demographic, socioeconomic, and physical–environmental characteristics (in 2001) of the four *groups of districts* presented in Figure 3.

Characteristic	High SES-Urban	High SES-Rural	Low SES-Rural	Low SES-Urban
Population	1,871,095	1,197,212	694,115	1,170,971
OECD	5.93	48.95	50.88	10.51
SES	2.06	1.56	−1.85	−3.62
% elderly	15.56	13.91	12.20	11.94
% low education	49.67	65.08	68.59	65.73
% unemployed	3.81	3.15	6.64	8.16
% singles	33.82	27.01	29.23	33.54
Summer T (°C)	18.4	17.4	17.6	17.5
Altitude (m a.s.l)	327	459	392	415
% impervious	29.82	4.45	4.40	13.42

**Table 3:** Spearman’s correlation matrix comparing the mean relative excess mortality due to CVDs on hot days (*DevCVD*) with independent variables used in the analysis (see Table 1 for description). Significant coefficients with *p*-value < 0.05 are in bold.

Independent Variable	DevCVD	SES	% Elderly	% low Education	% Unemployed	% Singles	OECD	Summer T (°C)	Altitude (m a.s.l.)
SES	0.01	1							
% elderly	0.14	**0.61**	1						
% low education	−0.20	**−0.52**	**−0.47**	1					
% unemployed	0.12	**−0.75**	**−0.53**	**0.33**	1				
% singles	0.13	**−0.38**	−0.04	**−0.24**	0.09	1			
OECD	−0.22	0.11	0.07	**0.47**	**−0.24**	**−0.56**	1		
Summer T (°C)	**0.37**	0.18	**0.38**	−0.19	0.10	−0.08	−0.19	1	
Altitude (m a.s.l.)	**−0.37**	−0.05	**−0.31**	**0.17**	−0.13	−0.09	**0.26**	**−0.92**	1
% impervious	**0.34**	0.05	0.12	−0.41	**0.24**	0.23	**−0.74**	**0.64**	**−0.66**

**Table 4:** Independent variables (see Table 1) chosen by the stepwise regression model as significantly related to the mean relative excess mortality due to CVDs on hot days (*DevCVD*) in individual districts. Regression coefficients are reported, along with their associated *p*-values in parentheses.

Independent Variable	DevCVD
SES	----
% elderly	----
% low education	----
% unemployed	----
% singles	----
OECD	−0.096 (0.043)
Summer T (°C)	2.922 (0.002)
% impervious	----
R^2^	0.191

**Table 5:** Linear regression models for mean relative excess mortality due to CVDs on hot days (*DevCVD*) against index of socioeconomic status (*SES*), percentage of rural population (*OECD* criterion), and mean summer temperature (*Summer T*) for districts with *low*, *intermediate*, and *high* socioeconomic status (*SES* class). Regression coefficients are reported, along with their associated *p*-values in parentheses. See Table 1 for description of variables examined. Significant regression coefficients with *p*-value < 0.05 are in bold.

SES Class	DevCVD *vs.* SES	DevCVD *vs.* Summer T (°C)	DevCVD *vs.* OECD	SES *vs.* OECD
Low	**−3.099 (0.034)**	**4.307 (0.025)**	**−0.193 (0.044)**	**0.041 (0.000)**
Intermediate	−3.399 (0.490)	**3.537 (0.008)**	−0.073 (0.407)	0.008 (0.293)
High	1.474 (0.842)	1.234 (0.181)	−0.164 (0.078)	−0.008 (0.347)
R^2^	0.086	0.160	0.107	0.878
